# A novel, highly efficient β-glucosidase with a cellulose-binding domain: characterization and properties of native and recombinant proteins

**DOI:** 10.1186/s13068-017-0946-2

**Published:** 2017-11-06

**Authors:** J. A. Méndez-Líter, J. Gil-Muñoz, M. Nieto-Domínguez, J. Barriuso, L. I. de Eugenio, M. J. Martínez

**Affiliations:** Department of Environmental Biology, Centro de Investigaciones Biológicas, CSIC, Ramiro de Maeztu 9, 28040 Madrid, Spain

**Keywords:** Fungi, Glycosyl hydrolases, *Pichia pastoris*, Carbohydrate binding modules, Saccharification, Brewers spent grain

## Abstract

**Background:**

Cellulose, the most abundant biopolymer on earth, is an alternative for fossil fuels as a renewable feedstock for the production of second-generation biofuels and other chemicals. The discovery of novel, highly efficient β-glucosidases remains as one of the major bottlenecks for cellulose degradation. In this context, the ascomycete *Talaromyces amestolkiae,* isolated from cereal samples, has been studied as a promising source for these enzymes.

**Results:**

BGL-2 is the major β-glucosidase secreted by this fungus in the presence of cellulosic inductors. This enzyme possesses a CBD (Cellulose Binding Domain), an unusual feature among this type of proteins. Besides, when growing on cellulose, the fungus produced two different *bgl*-*2* mRNAs that were cloned and expressed in *Pichia pastoris*. A complete recombinant protein (BGL-2*) and its truncated form, lacking CBD (BGL-2T*), have been purified, characterized and compared with the native enzyme (BGL-2). The three BGL-2 forms studied are highly stable in a wide pH range, but BGL-2T* showed an improved thermal stability at 50 °C after 72 h. Using *p*-nitrophenyl-β-d-glucopyranoside as a substrate, the steady-state kinetic characterization of the three proteins showed a similar *K*_*m*_ and *k*_*cat*_ for BGL-2 and BGL-2*, while the truncated protein displayed a threefold higher value for *k*_*cat*_. All tested BGL-2 enzymes were as well highly efficient using cellobiose and other short oligosaccharides as a substrate. In view of biotechnological applications, the recombinant *T. amestolkiae* enzymes in saccharification of brewers’ spent grain were studied, being comparable to commercial β-glucosidase cocktails.

**Conclusion:**

A new β-glucosidase from *T. amestolkiae* has been studied. The enzyme, containing a functional CBD, has been expressed in *P. pastoris*. The comparative analyses of the native protein and its recombinant forms, with and without CBD, suggest that they could be suitable tools for valorization of lignocellulosic biomass.

**Electronic supplementary material:**

The online version of this article (10.1186/s13068-017-0946-2) contains supplementary material, which is available to authorized users.

## Background

The current need of finding sustainable and renewable energy sources is making the degradation of lignocellulosic biomass to produce second-generation biofuels a blooming subject, since they may represent a viable alternative to fossil fuels [1, 2]. Lignocellulosic biomass degradation requires different steps: (i) pretreatment to improve polysaccharide accessibility, (ii) enzymatic hydrolysis of cellulose and hemicellulose and (iii) fermentation of free monosaccharides to ethanol [3]. Steam explosion is the most common pretreatment method used to disrupt plant cell wall components. Although very effective, the use of high pressures and temperatures generates undesirable compounds from sugars and lignin, which produce negative effects on enzymatic hydrolysis and fermentation [4].

The plant cell wall consists of three major components, whose proportions depend on the source of the lignocellulosic biomass: cellulose, hemicellulose, and lignin [5]. Nowadays, commercial enzymatic cocktails used for hydrolysis of lignocellulosic biomass contain cellulases, hemicellulases, and other complementary enzymes that facilitate the complete degradation of plant cell wall. Cellulose, the most abundant polymer on earth, is a polysaccharide composed of long linear chains of d-glucose linked by β-(1,4)glycosidic bonds. Besides being used for biofuels production, this polysaccharide is basic to a multitude of industrial processes like paper pulp and chemicals production [5]. Cellulose is enzymatically hydrolyzed by the combined action of endoglucanases (EGs) (EC 3.2.1.4), cellobiohydrolases (CBHs) (EC 3.2.1.91), and β-glucosidases (BGLs) (EC 3.2.1.21) [6]. The effect of CBHs and EGs on cellulose generates short soluble oligosaccharides, which are converted into glucose by β-glucosidases. These enzymes are members of the glycoside hydrolases (GHs) family and attack polysaccharides like cellulose in a relatively inefficient way, as their glycosidic bonds are often inaccessible to the active site of the enzymes. This is due to the low solubility of these polysaccharides and/or to their crystalline structures. A wide variety of glycoside hydrolases that degrade such insoluble substrates have structural domains known as carbohydrate binding modules (CBMs), for example, cellulose binding domains (CBDs), which are useful for the recognition and attachment of GHs to their substrates [7].

Although the synergistic action of all the cellulolytic activities is required to fully degrade cellulose, β-glucosidases are considered as the key enzymes for this process as they are indispensible for releasing free glucose. β-glucosidases are generally found in little proportion in commercial preparations, usually produced from *Trichoderma* and *Aspergillus* species [8]. Hence, many studies are focusing on finding robust β-glucosidases, since enzyme cocktails must be supplemented with this activity to increase the efficiency of cellulose saccharification [8].

Commercial β-glucosidases are typically obtained from filamentous fungi because fungal enzymes usually have a higher catalytic productivity/efficiency than other microorganisms. In the last years, the high cellulolytic potential of various *Penicillium* species and its perfect states, *Talaromyces* or *Eupenicillium*, has been reported [9, 10]. In this sense, a recent work revealed that *T. amestolkiae* produces several β-glucosidases in the presence of different carbon sources [11]. Here we report the purification, heterologous expression and biochemical characterization of a β-glucosidase produced by this fungus in the presence of cellulosic substrates. In addition, we evaluated its role in saccharification of brewers spent grain.

## Results and discussion

### Production, purification and properties of native BGL-2

*Talaromyces amestolkiae* has been recently described as a fungus producing a wide variety of cellulases. Different types of these enzymes were secreted as a function of the available carbon source. On cellulosic substrates, a β-glucosidase was abundantly produced. In this work, this novel β-glucosidase has been purified from 8-day-old *T. amestolkiae* cultures growing in Mandels medium containing 1% Avicel, when maximal BGL activity levels were reached. For purification, three consecutive chromatographic steps were needed. The filtered, concentrated and dialyzed crude was first loaded on a HiTrap Capto Adhere cartridge, a strong anion exchanger with multimodal functionality. Two peaks containing BGL activity were recovered indicating that, in these conditions, the fungus produced at least two β-glucosidases. This finding agrees with the results from a proteomic analysis of the secretome released in Avicel cultures of this fungus, which disclosed the production of different β-glucosidases [11]. Proteins from peak 2 (containing around 79% of initial BGL activity), were concentrated and loaded in a Mono Q column. After this chromatographic stage, again, two peaks with BGL activity were separated. The first peak, containing 80% of total BGL activity of this step, was concentrated and subjected to size exclusion chromatography in Superdex 75 HR 10/30 to complete the protein purification. This protein, denominated as BGL-2, was purified around fourfold with a final yield of 6.3% (Table 1).

**Table 1:** Purification yield of native and recombinant BGL-2 isoforms

Step	Total protein (mg)	Total activity (U)	Specific activity (U/mg)	Yield (%)
BGL-2				
Crude extracts	53.4	1039.7	19.5	100
HiTrap Capto Adhere	15.8	795.6	50.4	76.5
Mono Q 5/50	7.6	596.4	78.5	57.3
Superdex 75 HR 10/30	0.8	66.1	82.6	6.3
BGL-2*				
Crude extracts	25.1	300.1	11.9	100
HiTrap Capto Adhere	7.64	126.5	16.5	42.2
BGL-2T*				
Crude extracts	19.9	436.1	21.8	100
HiTrap Capto Adhere	3.6	128.8	35.4	29.5

**Fig. 1:**
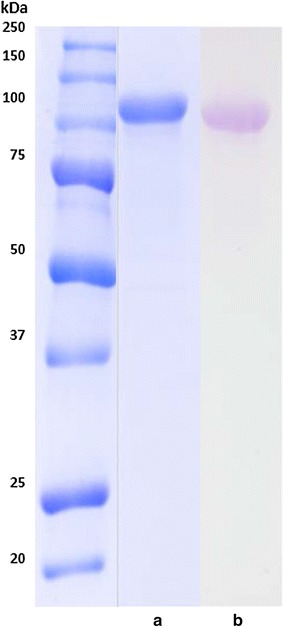
SDS-PAGE **a** and PAS staining on SDS-PAGE **b** of BGL-2 from *T. amestolkiae*

The molecular mass of BGL-2, estimated by SDS-PAGE, was around 100 kDa (Fig. 1a). Besides, although enzymatic *N*- and *O*-deglycosylation of BGL-2 did not alter its molecular mass, PAS staining showed a purple BGL-2 band, indicating its glycoprotein nature (Fig. 1b). This result suggests that BGL-2 may be *O*-glycosylated since enzymatic *O*-deglycosylation is not very efficient for fungal glycoproteins due to the heterogeneity of this post-translational modification, oppositely to *N*-deglycosylases that are usually very efficient because *N*-glycosylation is evolutionary conserved [12].

### Sequence analysis and homology modeling of BGL-2

The peptide mass fingerprinting of this protein evidenced internal peptides that showed high similarities with a hypothetical β-glucosidase (XP_002149046) from *Talaromyces marneffei* ATCC 18224. Using TBLASTN, the sequence of these peptides was used to identify the *bgl2* gene in the genome of *T. amestolkiae,* deposited in the NCBI database with accession number of MIKG00000000.

Analysis of *bgl*-*2* gene indicated that this protein belongs to the GH3 family. Surprisingly, a sequence compatible with the presence of a CBD was found. This region is common in cellobiohydrolases and endoglucanases [13], but very rare in a β-glucosidase. The CBD was linked to the catalytic domain through a serine–threonine rich region that would act as a flexible connector between both regions, as reported for *Trichoderma reesei* cellobiohydrolase I [14]. The ability to bind cellulose has only been described for the β-glucosidase of *Phanerochaete chrysosporium* [15], which has a CBD in its N-terminal domain, but this enzyme was finally identified as a glucan-1,3-β-glucosidase [16], with much more activity on laminaribiose and laminarin than on cellobiose. Hence, *T. amestolkiae* BGL-2 is the first 1,4-β-glucosidase where a CBD has been characterized.

Intron and exon identification was performed by comparison with transcriptomic data from the most similar sequences (XM_002485083 and XM_002149010) using BlastN. It was concluded that *bgl2* gene is interrupted by three introns, with a sequence of 2460 bp, coding for 819 amino acids (GenBank Accession Number: KM393203). Intron prediction was confirmed after RNA extraction and cDNA production.

The information gathered in the databases on the crystal structures of fungal β-glucosidases from the GH3 family is very limited. Only four GH3 crystal structures are available in Protein Data Bank (PDB), three from *Hypocrea jecorina* (synonym of *Trichoderma reesei*) (4I8D, 3ZZ1 and 3ZYZ) [17] and one from *Aspergillus aculeatus* (4IIB) [18]. As in BGL-2, the catalytic domain of these proteins is divided into three subdomains: a N-terminal domain with TIM (α/β) 8 barrel form, a C-terminal domain with α/β sandwich, and a fibronectin III-like domain, with unknown role and possible implications in thermal stability [18, 19]. The model structure of *T. amestolkiae* BGL-2 was obtained using structures 3ZYZ and 4I8D from PDB as templates, which showed an identity of 64.2%. QMean value in both cases was − 3, indicating that the model was well adjusted to the experimental structures.

**Fig. 2:**
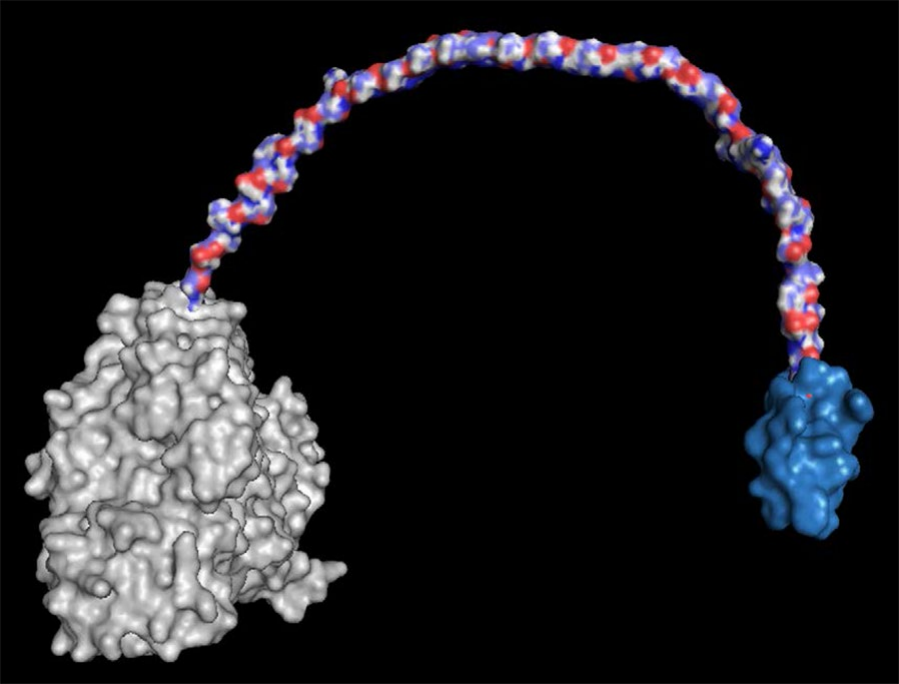
Molecular surface model of BGL-2 from *T. amestolkiae*. The catalytic domain (grey), the connector (blue, red and white) and the CBD (blue) are depicted

The CBD of BGL-2 was modeled separately since the fungal β-glucosidases of *H. jecorina* lack this domain. The model that best adjusted (QMean − 0.51) came from a CBH of this fungus (PDB:1CBH). Finally, the modeling of the linker between the catalytic domain and CBD was performed manually because there is no such crystallized structure deposited, probably due to its high flexibility (Fig. 2).

**Fig. 3:**
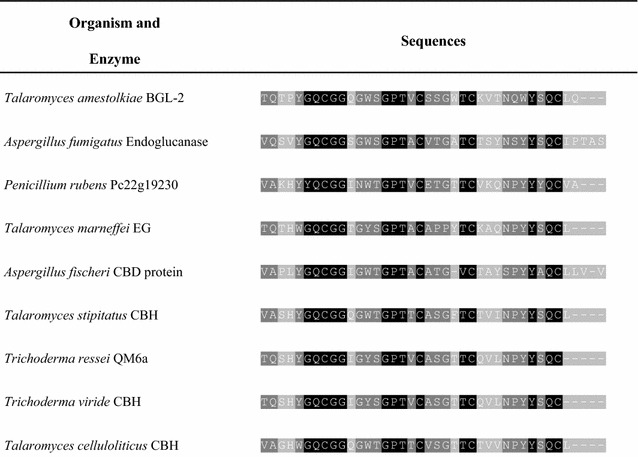
Sequence alignment of the CBD region of *T. amestolkiae* BGL-2 with other CBDs reported in different fungal cellulases using the T-coffee alignment program. The accession numbers of the compared enzymes are: XP_748707.1 (*A. fumigatus*), XP_002565826.1 (*P. rubens)*, XP_002152969.1 (*T. marneffei*), XP_001262453.1 (*A. fischeri*), and XP_002484839.1 (*T. stipitatus*), XP_006969224.1 (*T. reesei*), AAQ76092.1 (*T. viride*), and GAM33347.1 (*T. celluloliticus*). Black color shows the conserved residues in all cases. Dark grey shows the amino acids with 1 or 2 variations. Light grey indicates the residues with 3 or more variations between the sequences

The BGL-2 CBD sequence was compared with CBMs reported for other cellulases using the protein–protein Blast database (BlastP), indicating that it belongs to family 1 (CBM1) since it had most of the conserved amino acids characteristic of this family (Fig. 3). With some of the best matches, a sequence alignment using T-coffee program was performed.

### Heterologous expression of *bgl2*

PCR fragments containing *bgl2* cDNA obtained by reverse transcription were purified and cloned into pPIC9. Unexpectedly, two different sequences were obtained: one with the expected size for the fungal gene sequence after excision of the three predicted introns (2460 bp length) and another 65 bp longer (2525 bp), which had retained the third intron, maybe due to an alternative or wrong RNA processing. Clones with this sequence will produce a truncated form of BGL-2 (without CBD) since this intron contains a stop codon upstream the CBD coding region. Thus, two constructs were isolated: I) a fragment that will produce a truncated form of *bgl2* without CBD, named BGL-2T*, with a coding sequence of 2202 bp and 734 amino acids; and II), the fragment that will generate the complete form of *bgl2*, including the CBD, that will be named BGL-2*, with a coding sequence of 2460 bp and 820 amino acids (Additional file 1: Figure S1). This misprocessing of mRNA in *bgl2* gene could be related to an alternative splicing phenomenon. In fact, alternative splicing mechanisms have been described in some cellobiohydrolases from *P. chrysosporium*, in which selective transcription of CBD occurs depending on the carbon source [20]. As other filamentous fungi, *T. amestolkiae* exhibits the property of expressing different isoforms of cellulases depending on the culture conditions or carbon sources.

The pPIC9 vectors containing both cDNA fragments encoding BGL-2 and its truncated form BGL-2T* were transformed into *P. pastoris* KM71 strain. Transformants selected in YNB-His medium were plated in YPM to detect β-glucosidase activity after MUG-agar incubation, as described in “Methods” section. The transformants that produced higher fluorescence were chosen as potential enzyme producers because they were able to hydrolyze MUG efficiently.

### Production of BGL-2* and BGL-2T* in *Pichia pastoris* and purification of the recombinant enzymes

**Fig. 4:**
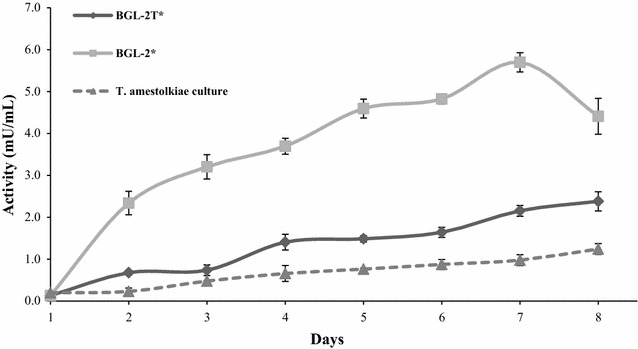
β-glucosidase activity secreted by *T. amestolkiae* in the presence of Avicel, and BGL-2* and BGL-2T* production in *P. pastoris*

Figure 4 shows that recombinant *P. pastoris* yeast strains, which produce either the complete, or the truncated form of BGL-2, secreted higher β-glucosidase activity levels than *T. amestolkiae* growing in Mandels medium with Avicel as carbon source (fivefold and twofold, respectively). Although the time needed for optimal secretion was similar, the higher production levels and the few extracellular proteins secreted by *P. pastoris* strains facilitated its further purification.

Both recombinant proteins were purified in a single chromatographic step using a HiTrap Capto Adhere cartridge, with a yield of 42 and 29% for BGL-2* and BGL-2T*, respectively, which represent an increased yield of sevenfold and fivefold compared with the native protein.

### Comparative properties of native and recombinant BGL-2 proteins

Molecular mass of BGL-2, BGL-2* and BGL-2T* were studied by MALDI-TOF MS and size exclusion chromatography. All BGL-2 forms are monomeric enzymes, with an accurate molecular mass of 97.2, 102.1 and 80.8 kDa for BGL-2, BGL-2* and BGL-2T*, respectively (Additional file 1: Figure S2). The differences found between theoretical (83.1 kDa for the complete enzymes and 74.4 kDa for the truncated BGL-2) and empirical molecular masses can be attributed to protein glycosylation: 14.7%, for BGL-2, 18.7% for BGL-2* and 8% for BGL-2T*. It is noticeable that the truncated form without CBD is fairly less glycosylated than the proteins with this domain. This could be explained in terms of protein composition, since BGL-2T* also lacks the linker region, where 39 possible glycosylation sites have been identified (Additional file 1: Figure S3). Linkers usually are strongly glycosylated, preventing proteolysis of this region [21, 22].

Isoelectrofocusing indicated that the isoelectric point of purified β-glucosidases were 5.6, 7.4 and 5.2 for BGL-2, BGL2* and BGL-2T*, respectively. The major difference was found between BGL-2 and its recombinant form. The theoretical IEF value predicted from the BGL-2T* sequence was 4.89, similar to the one obtained experimentally. However, for BGL-2* the theoretical value was 5.06. These differences between theoretical and experimental isoelectric points of the complete forms of the native and the recombinant enzyme, could be attributed to their different glycosylation patterns, that can affect the isoelectric point of a given protein [23].

**Fig. 5:**
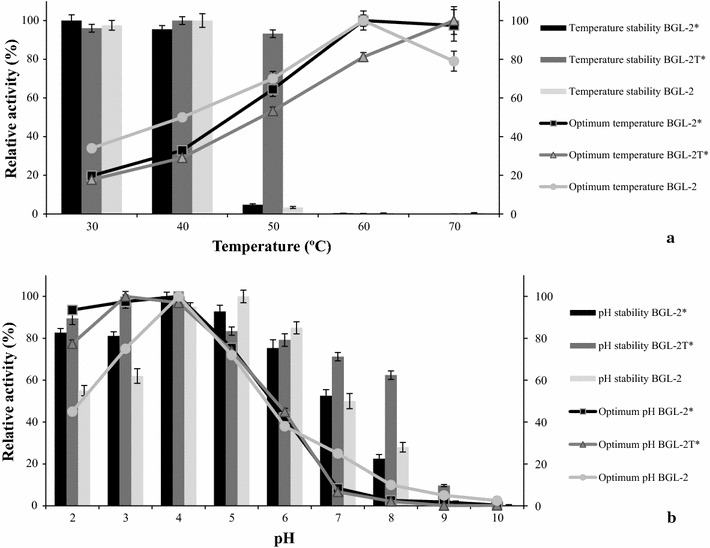
Effect of temperature **a** and pH **b** on BGL-2 enzyme activity. Lines indicate the optimum temperature or pH for enzyme activity; bars show its 72 h-stability in a range of temperatures from 30–70 °C or pH from 2 to 10

Temperature and pH are crucial factors for the enzymatic hydrolysis of lignocellulosic biomass degradation. All BGL-2 isoforms studied were stable up to 40 °C after 72 h. Remarkably, the lack of CBD increased the temperature stability of BGL-2T* to 50 °C after 3 days (Fig. 5a). BGL-2T* also was more stable in the most acidic and basic pH assayed (Fig. 5b). Optimal activity of BGL-2T* was obtained between pH 3–4 and 70 °C, while both, BGL-2 and BGL-2* showed their highest activity at a similar pH but at 60 °C. These results suggest that all BGL-2 forms studied could be useful for biotechnological applications such as 2G ethanol production from lignocellulosic wastes, especially those acid-pretreated [24]. Comparing with the data obtained for other native β-glucosidases, all BGL-2 forms had superior optimal temperatures [25] and were stable at broader pH ranges [26, 27]. The recombinant β-glucosidases from *Thermoascus aurantiacus* [28], *Aspergillus fumigatus* [29], *Myceliophthora thermophila* [30], *Neosartorya fischeri* [31], *Penicillium funiculosum* [32] and *Neurospora crassa* [33], expressed in *P. pastoris*, had optimum temperatures similar to BGL-2*, between 60 and 70 °C, with the exception of *N. crassa*, whose optimal temperature is 80 °C. The optimal pH for these proteins was between 5 and 6, whereas the optimum pH of the recombinant BGL-2 forms is more acidic.

The circular dichroism analysis of native and recombinant proteins revealed that all BGL-2 forms have a typical spectrum of α + β folded structures. The native and recombinant full-length proteins had very similar spectra, confirming their common secondary structure. However, the spectrum of BGL-2T* lacked the minimum at 208 nm typical of proteins with CBD (Additional file 1: Figure S4). This study evidenced that the two forms of the protein do not have the same secondary structure, suggesting that BGL-2T* could have a slightly different folding. This may be one of the reasons for its increased stability to temperature and pH. A recent report has shown the negative effect of excessive *O*-glycosylation on the pH stability of a recombinant β-glucosidase from *Talaromyces leycettanus* [34], which is in good agreement with the lower glycosylation degree and higher stability determined for BGL-2T* against those of the full-length proteins. However, the improved stability of BGL-2T* could be also explained in terms of protein composition: an endo-β-1,4-glucanase from *Bacillus subtilis* JA18 showed increased thermal stability and catalytic efficiency after CBD depletion [35]. These authors postulated that the increased stability could be related to an enhanced refolding after thermal stress in the absence of CBD. In this sense, the deletion of CBD and its linker could result in a more compact protein, with a larger fractional polar surface, increasing hydrogen bonding density to water [36].

### Cellulose binding assay

To determine whether the CBD had the ability to bind cellulose, Avicel adsorption tests were performed with native and recombinant BGL-2, with and without CBD.

**Fig. 6:**
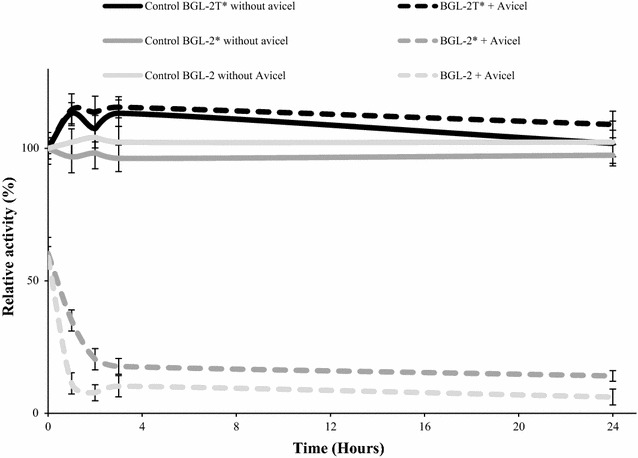
Cellulose-binding assays. The three BGL-2 forms were incubated with Avicel for 24 h at 4 °C and residual β-glucosidase activity was measured in the supernatants

The results showed that native BGL-2 and BGL-2* quickly bind to Avicel, decreasing β-glucosidase activity in the supernatants, while no activity changes were observed in the supernatants of the assays with the truncated protein (Fig. 6). In addition, the interaction between Avicel and the proteins’ CBD was strong and stable after 24 h, which suggests that this domain is functional, and could play an important role in binding to natural cellulosic substrates. Finally we have examined the CBD binding ability to other polysaccharides like xylan and chitin. The results showed that the CBD of BGL-2 binds specifically to cellulose (Additional file 1: Figure S5).

### Substrate specificity of the native and recombinant BGL-2 forms

The three forms of BGL-2 hydrolyzed *p*NPG, *o*NPG and cellooligosaccharides from cellobiose to cellohexaose with different efficiency. As a general rule, the recombinant BGL-2 enzymes were more active and showed higher affinity towards all substrates tested. More specifically, BGL-2T* presented a superior catalytic efficiency towards all substrates but cellopentaose and cellohexaose (Table 2). In detail, it can be seen that the two recombinant proteins showed a better affinity for the longer cellooligosaccharides. The different catalytic efficiency of BGL-2* and BGL-2T* on cellooligosaccharides of different lengths could be related to the presence or absence of the CBD, since the role of CBMs in binding to cellotetraose or cellohexaose has already been demonstrated [37, 38]. The higher catalytic efficiency of BGL-2T* on the shorter substrates could also be explained considering that the CBD could interfere with the access of small oligosaccharides to the catalytic site. On the other hand, the importance of the CBD to bind the enzyme to longer substrates, like cellopentaose and cellohexaose, might favor their hydrolysis rate, being responsible for the improved catalytic efficiency of these molecules by BGL-2*. The lower effect of the native BGL-2 on all the cellooligosaccharides assayed could be due to some negative effect on the integrity of the purified enzyme across its multistep purification process.

**Table 2:** Kinetic parameters of all BGL-2 isoforms against different substrates

Substrate	*K*_m_ (mM)			k_cat_ (s^−1^)			k_cat_/*K*_m_ (mM^−1^ s^−1^)		
	BGL-2	BGL-2*	BGL-2T*	BGL-2	BGL-2*	BGL-2T*	BGL-2	BGL-2*	BGL-2T*
*p*NPG	0.41 ± 0.02	0.19 ± 0.01	0.34 ± 0.02	485	444	874	1167	2243	2563
*o*NPG	0.86 ± 0.04	1.48 ± 0.02	0.67 ± 0.04	174	59	217	201	39	323
Cellobiose	1.21 ± 0.04	1.11 ± 0.02	0.91 ± 0.05	303	630	569	249	567	619
Cellotriose	1.37 ± 0.05	1.87 ± 0.13	1.42 ± 0.06	272	617	716	198	329	502
Cellotetraose	1.68 ± 0.04	0.92 ± 0.01	0.49 ± 0.03	313	580	402	185	629	813
Cellopentaose	1.28 ± 0.02	0.71 ± 0.03	0.68 ± 0.01	309	671	482	239	936	701
Cellohexaose	1.01 ± 0.04	0.51 ± 0.01	0.49 ± 0.03	313	405	359	307	794	720

The kinetic parameters determined for all BGL-2 isoforms, and especially the recombinant enzymes, show that these enzymes are among the most efficient BGLs discovered up to now (Additional file 1: Table S1). However, very few studies describe high BGL activities on natural substrates as cellobiose or cellooligosaccharides, despite these are their typical substrates in saccharification processes [39]. All the BGL-2 isoforms analyzed hydrolyzed cellooligosaccharides C2–C6 more efficiently than all BGL studied so far (Table 3). These results put these enzymes in the forefront of the known β-glucosidases for the saccharification of these natural substrates, which is highly relevant from a biotechnological perspective.

**Table 3:** Comparison of cata lytic efficiencies against cellooligosaccharides (C2–C6) of fungal β-glucosidases

Organism and reference	Enzyme	Cellobiose	Cellotriose		Cellotetraose		Cellopentaose		Cellohexaose	
		*K*_cat_ */K*_*m*_	*K*_m_	*V*_max_	*K*_m_	*V*_max_	*K*_m_	*V*_max_	*K*_m_	*V*_max_
Native BGLs										
*Trichoderma reesei* [50]	Cel3A	37.3	0.2	38.0		41.0				
	Cel3B	21.6	0.3	36.0		36.0				
	Cel1A	70.6	1.1	18.0		2.6				
Metagenome from compost [51]	Td2f2	1.6	3.1	10.1	1.53	7.8	8.4	7.9		
*T. thermosaccharolyticum* [52]		13.3								
*Penicillium purpurogenum* [40]				111.0		96.0		68.4		
BGLs expressed in *P. pastoris*										
*Aspergillus fumigatus* [29]	rBgl3	52.1								
*Penicillium funiculosum* [32]	rBgl4	3610.4		59.4		39.3		32.7		23.70
*Neurospora crassa* [33]	BGL2			12.3		9.4				
*Myceliophthora thermophila* [30]	MtBgl3a	17.4								
BGLs of this work										
*T. amestolkiae*	BGL-2	249.1	1.37	167.8	1.69	193.1	1.2	190.7	1.0	193.2
	BGL-2*	567.1	1.88	363.2	0.92	341.5	0.7	394.8	0.5	238.7
	BGL-2T	619.1	1.43	532.2	0.50	298.9	0.6	358.1	0.5	266.9

A set of aryl glycosides and disaccharides were also assayed to test the enzymes’ specificity. Residual activity levels were detected towards 4-nitrophenyl β-d-xylopyranoside and 4-nitrophenyl α-d-glucopyranoside, and no activity was observed against 4-nitrophenyl β-d-galactopyranoside, 4-nitrophenyl α-d-galactopyranoside, 4-nitrophenyl α-l-rhamnopyranoside, 4-nitrophenyl β-d-fucopyranoside, maltose, lactose or sucrose. Remarkably, the three enzymes were quite active towards laminaribiose and gentiobiose (data not shown).

In addition, all BGL-2 isoforms were capable of releasing reducing sugars from Avicel, carboxymethyl cellulose (CMC), and beechwood xylan (Table 4). This is quite unusual since polysaccharides are typically not degraded by these enzymes. It was noticeable that, unlike for small carbohydrates, the native enzyme and the recombinant BGL-2 had similar catalytic activity. The full-length BGL-2 showed slightly more specific activity against these polymers than the truncated enzyme. Hence, although the CBD is not strictly needed for BGL-2 to hydrolyze small soluble substrates, its presence increases polysaccharide conversion, probably by improving the enzyme binding. The activity over Avicel of a fungal β-glucosidase from *A. fumigatus,* expressed in *P. pastoris*, was significantly lower (1.7 U/mg) [29] than that detected for the BGL-2s from *T. amestolkiae*. On the contrary, a β-glucosidase from *P. purpurogenum* [40] showed higher specific activity, but it was inactive against other polysaccharides like xylan or CMC. In general, most investigations report that BGLs have no activity on polysaccharides. The ability of BGL-2 to hydrolyze these substrates shows its versatility to be used in industrial applications.

**Table 4:** Specific activity of BGL-2, BGL-2* and BGL-2T* against different polysaccharides

Substrate	Specific activity (U/mg)				
	BGL-2	BGL-2*	BGL-2T*	Celluclast 1.5 L	Inactivated enzyme^a^
Avicel	11.2 ± 0.5	15.8 ± 0.4	6.5 ± 0.3	4.4 ± 0.2	0.51 ± 0.1
CMC	9.1 ± 0.2	10.0 ± 0.4	5.4 ± 0.4	11.3 ± 0.4	0.88 ± 0.12
Xylan	21.6 ± 0.2	18.3 ± 0.3	14.2 ± 0.2	3.5 ± 0.3	0.74 ± 0.12

^a^Boiled 20 min

### Brewers spent grain saccharification

The enzymatic conversion of cellulose to glucose is a crucial step in the production of bioethanol from lignocellulosic biomass. Since commercial cellulolytic cocktails based on enzymes from *T. reesei*, like celluclast, are usually deficient in β-glucosidase activity, they are supplemented with this enzymatic activity for lignocellulosic biomass treatment. β-glucosidases from *Penicillium* sp. or *Talaromyces* sp. have successfully been applied for saccharification of cellulosic materials, being more efficient than *Trichoderma* sp. enzyme preparations, since they have higher β-glucosidase levels [41]. For saccharification of brewers spent grain, the synergistic effect of Celluclast 1.5 L and a β-glucosidase-rich supplement (the native or recombinant isoforms of *T. amestolkiae* BGL-2 or the commercial preparation NS-50010) were evaluated.

**Fig. 7:**
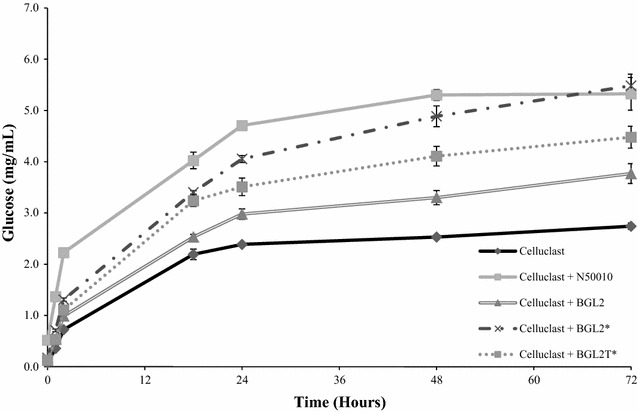
Glucose yield from saccharification of brewers spent grain. Addition of one of the three BGL-2 enzymes or NS-50010 to Celluclast 1.5 L improved substrate degradation

The results shown in Fig. 7 indicate that supplementation with any of the BGL-2 forms enhanced the saccharification of brewers spent grain, than using only celluclast (8% with BGL-2, 32% with BGL-2* and 24% for BGL-2T). The minor effect of the native *T. amestolkiae* protein could be related to their kinetic constants, slightly worse than those determined for the recombinant proteins against the substrates assayed. The differences found when Celluclast was supplemented with BGL-2T*, could indicate that the CBD could have a relevant role in binding cellulose in natural substrates increasing its degradation efficiency. Interestingly, the supplementation with BGL-2* showed a similar yield than with NS-50010. This is remarkable since BGL-2* is a purified protein, whereas NS-50010 is an enzyme cocktail, containing other cellulolytic and hemicellulolytic activities which could promote the yield of the saccharification process. Hence, this results suggest that both recombinant *T. amestolkiae* β-glucosidases, especially BGL-2*, could be suitable proteins in the valorization process of lignocellulosic biomass.

## Conclusions

β-glucosidases are very versatile enzymes that play an essential role in the enzymatic hydrolysis of plant biomass for the production of 2G biofuels. In the present work, BGL-2 from *T. amestolkiae* was discovered, purified and characterized, standing out for being the first 1,4-β-glucosidase with a functional cellulose binding domain, similar to others found in CBHs and EGs. Besides, an isoform without CBD was also isolated and characterized. The work has revealed the high potential of the native BGL-2 and its recombinant forms, with and without CBD, to be applied for the saccharification of plant biomass. The complete characterization of the cellulolytic system of this fungus is currently being carried out, with special interest in the purification of other β-glucosidases produced by this strain, to evaluate their properties and features to be used in different biotechnological applications.

## Methods

### Microorganism and culture conditions

The fungus used in this work, isolated from cereal samples by the group of Dr. Covadonga Vazquez (Department of Microbiology, Faculty of Biology, Complutense University of Madrid), was identified and included in the Collection of the Institute Jaime Ferrán of Microbiology (IJFM) with the number A795. The fungus was grown in potato dextrose agar (PDA) for 5 days at 28 °C. Spore suspensions were obtained by placing 1 cm^2^ agar slants in 5 mL of a 1% NaCl solution, and 0.1% of Tween 80. The mixture was shaken and 200 µl were used to inoculate 250 mL flasks with 50 mL of CSS medium (40 g/L glucose, 0.4 g/L FeSO_4_·7H_2_O, 9 g/L (NH_4_)_2_SO_4_, 4 g/L K_2_HPO_4_, 26.3 g/L corn steep solid, 7 g/L CaCO_3_, and 2.8 mL/L soybean oil). The culture was incubated at 28 °C and 250 rpm for 5 days.

To grow *T. amestolkiae* for enzyme production, 2 mL from a CSS culture were inoculated in Mandels medium [42], composed by: 2.0 g/L KH_2_PO_4_, 1.3 g/L (NH_4_)_2_SO_4_, 0.3 g/L urea, 0.3 g/L MgSO_4_·7H_2_O, 0.3 g/L CaCl_2_, 5 mg/L FeSO_4_·7H_2_O, 1.6 mg/L MnSO_4_·H_2_O, 1.4 mg/L ZnSO_4_·7H_2_O, and 1 g/L Bacto peptone. The pH was adjusted to 4.5. This medium was supplemented with 1% Avicel (microcrystalline cellulose) as carbon source and inducer of cellulolytic activities. The inoculated flasks were incubated at 28 °C and 250 rpm for 8 days, taking daily culture samples for analytical determinations.

*Escherichia coli* DH5α (Invitrogen) was used for plasmid propagation. It was grown in LB medium (10 g/L tryptone, 5 g/L yeast extract, 10 g/L NaCl, and 15 g/L agar), at 37 °C, overnight. For the growth and selection of ampicillin resistant transformants, this antibiotic was included in the LB medium, as its sodium salt, in a final concentration of 100 mg/L, and bacteria were grown overnight at 37 °C.

The heterologous expression of β-glucosidases were performed using *P. pastoris* KM71 strain (Invitrogen) as a host, which was preserved in YPD medium plates (10 g/L yeast extract, 20 g/L peptone, 20 g/L glucose and 10 g/L of agar). The transformants were screened in a selective medium, YNB-His (20 g/L glucose, 6.7 g/L YNB, 1.92 g/L Yeast synthetic drop-out medium supplements without histidine (Sigma-Aldrich), and 10 g/L agar), and cultured 72 h at 28 °C. Finally, for recombinant protein production, YEPS medium was used (20 g/L peptone, 10 g/L yeast extract, 10 g/L sorbitol, and 100 mM potassium phosphate buffer, pH 6), with daily addition of 6.5 mL/L of methanol as inducer. Cultures were incubated for 7 days, at 28 °C and 250 rpm, taking samples daily to measure protein production. All experiments were performed by triplicates.

### Purification and characterization of native and recombinant proteins

All protein purifications were performed using an AKTA Purifier HPLC system (GE Healthcare Life Sciences). The native BGL-2 protein from *T. amestolkiae* was purified from protein extracts obtained after fungus growth in Mandels medium with 1% Avicel after 8 days of incubation, when maximal activity was detected. Culture supernatants were filtered, concentrated, and dialyzed though a 10 kDa cutoff membrane against 10 mM sodium phosphate pH 6, which is the buffer used across all the chromatographic steps required to purify the protein. The concentrate crude was first applied onto a Capto Adhere HiTrap cartridge (GE Healthcare Life Sciences) equilibrated with buffer using a 1 mL/min flow. Peak 1 eluted during the NaCl gradient (from 0 to 0.5 M), while peak 2 eluted once the gradient had finished, during reequilibration of the cartridge in the starting buffer. Fractions with high β-glucosidase activity were pooled, concentrated and tested for purity. The second purification step involved anion-exchange chromatography in a high resolution Mono Q column (GE Healthcare Life Sciences) equilibrated with 10 mM sodium phosphate buffer, pH 6, at a flow rate of 0.8 mL/min. The retained proteins were eluted with a linear NaCl gradient (0–0.25 M over 50 min). Then the column was washed with 1 M NaCl for 7 min and equilibrated in the initial conditions. As above, the fractions with BGL activity were pooled, concentrated, and tested for purity. A third step of size exclusion chromatography on Superdex 75 HR 10/30 (GE Healthcare Life Sciences) was required to achieve BGL-2 purification. The column was equilibrated and the proteins eluted in the same buffer with 100 mM NaCl to avoid unspecific interactions, at a 0.3 mL/min flow.

In the case of the recombinant enzymes, the purification was achieved in a single chromatographic step. *P. pastoris* cultures producing the maximal BGL activity (8 days old) were centrifuged and the supernatant was then concentrated, dialyzed and applied to a Capto Adhere HiTrap cartridge (GE Healthcare Life Sciences) in the same conditions as native protein. Once the NaCl gradient (from 0 to 0.5 M) was finished and the cartridge reequilibrated, a peak with pure BGL-2 isoforms was obtained.

In all chromatographic steps, protein concentration (A_280 nm_) and β-glucosidase activity, using *p*-nitrophenyl-β-d-glucopyranoside (*p*NPG) as substrate, were measured. Protein homogeneity was checked after each purification step by 10% SDS-PAGE staining with Coomassie brilliant blue R-250. The approximate molecular mass of the proteins was calculated by this technique comparing the migration of the bands with those of the molecular weight markers provided by Bio-Rad. The accurate determination of the protein molecular mass was done by MALDI-TOF.

### Identification of BGL-2 by peptide mass fingerprinting

Purified proteins were loaded on a 10% SDS-PAGE gel that, after the electrophoretic run, was stained with Sypro Ruby. Small pieces from the protein band were excised and digested with trypsin in accordance with the protocol reported by Shevchenko et al. [43]. MALDI-MS and MALDI-MS/MS data were obtained automatically in a mass spectrometer MALDI-TOF/TOF Autoflex III (Bruker Daltonics) equipped with a laser and a Smartbeam LIFT-MS/MS device. The data obtained were combined using the 3.0 BioTools (Bruker Daltonics) software, and mass values from trypsin, keratin, matrix or sodium adducts were removed. Data analysis was performed against the NCBInr database (National Center for Biotechnology Information non-redundant) with the 2.3 MASCOT search engine (Matrix Science). Relevant search parameters were set as follows: trypsin as enzyme, carbamidomethylation of cysteines as fixed modification, methionine oxidation as variable modification, 1 missed cleavage allowed, peptide tolerance of 50 ppm, and MS/MS tolerance of 0.5 Da. Protein scores greater than 75 were considered significant.

### Nucleic acid isolation, PCR and RT-PCR methods

Mycelium from cultures grown in Mandels medium was used for genomic DNA and RNA extraction after filtration of 8 days old cultures with 0.8 μm nitrocellulose filters. DNA and RNA extraction were carried out using DNeasy Plant Mini Kit and RNeasy Plant Mini Kit (Qiagen) respectively, according to the manufacturer instructions. The extracted nucleic acids were quantified using a NanoDrop ND-100 (Thermo Scientific).

Isolated transcripts were converted to cDNA using the Superscript II Reverse Transcriptase RT-PCR kit (Invitrogen) using 50 µM random hexamers. PCR amplifications were performed in a thermocycler Mastercycler pro S (Eppendorf) using genomic DNA as template. Reaction mixtures were subjected to an initial denaturation at 95 °C for 5 min, followed by 36 cycles of amplification of 95 °C of denaturation for 45 s, 50 °C for 45 s of primer annealing step, and 72 °C for 3 min of elongation, followed by a final extension step at 72 °C for 10 min. The amplified sequences were separated in a 0.8% (w/v) agarose electrophoresis gel stained with GelRed, cut out, and purified using a QIAquick gel extraction kit (Qiagen). For amplifying *bgl2* gene from cDNA, two primers with restriction sites were used: *Fw*-*BGL2SnaBI* (5′TACGTACAGTCAGCTTCTTGGTCCGCAG3′) and *RV*-*NotIBGL2* (5′GCGGCCGCCTATTGTAGGCATTGAGAATACCACTGATTC3′). PCR reaction mixtures contained: 1 × PCR Buffer; 1.5 mM MgCl_2_; 0.25 mM dNTPs; 0.25 mM forward and reverse primers; 100 ng of DNA template; and 0.05 U/mL of Taq polymerase.

### Plasmid construction, *E. coli* propagation and heterologous expression

For *bgl2* heterologous expression, the amplification product was digested with *SnaBI* and *NotI* restriction enzymes, and was ligated to pPIC9 vector, previously digested with the same enzymes, using T4 phage ligase (Promega). Plasmids which contain *bgl2* wild type and *bgl2* retaining the third intron were built and sequenced using the BigDye Terminator v3.1 cycle sequencing kit and the automated ABI Prism 3730 DNA sequencer, in Secugen (Madrid, Spain).

The plasmids obtained from *E. coli* positive clones were linearized with *Sal*I, and transformed into KM71 *P. pastoris* strain by the lithium chloride transformation method, according to the *Pichia* expression kit (Invitrogen). The transformants were screened by histidine auxotrophy in YNB-His medium.

Selected clones on YNB-His plates were cultured on YPM medium (10 g/L yeast extract, 10 g/L peptone, and 10 g/L of agar; after autoclaving the medium, 15 mL/L sterile methanol was added), to identify positive transformants. The yeasts were cultured 24 h at 28 °C. The plates were covered with a solution of 50 mM 4-methylumbelliferyl-β-d-glucopyranoside (MUG) in 100 mM sodium acetate buffer and 0.8% agar. After solidifying, the plate was introduced in an oven at 50 °C for 20 min, and positive clones were revealed in a transilluminator Bio Rad Gel Doc XR. Clones with β-glucosidase activity hydrolyze MUG, releasing methylumbelliferyl, which is fluorescent at UV wavelength [32].

### Physicochemical properties and homology modeling

The accurate molecular mass and homogeneity of the pure enzyme were analyzed by matrix-assisted laser desorption ionization–time of flight mass spectrometry (MALDI-TOF) using an Autoflex III instrument (Bruker Daltonics).

To know if the β-glucosidases of *T. amestolkiae* were glycoproteins, the PAS (Periodic Acid–Schiff) staining method was used using the Schiff reagent (Sigma), following the manufacturer instructions. The glycoproteins exhibit visible red–purple bands after approximately 20 min. *N*- and *O*-deglycosylation assays of purified proteins were carried out using Endoglycosidase *H* or *O*-glycosidase (Roche), according to the manufacturer instructions. Differences in molecular mass before and after deglycosylation were analyzed by SDS-PAGE electrophoresis, as described above. The possible glycosylation sites of BGL-2 were also analyzed with NetNGlyc 1.0 Server (http://www.cbs.dtu.dk/services/NetNGlyc/). Protein sequence was also submitted to the SignalP 4.1 server for identifying and locating the signal peptide, which was excluded from the mass prediction.

The isoelectric point of the native and recombinant proteins was determined by isoelectrofocusing on 5% polyacrylamide gels using pH 3–10 ampholytes (GE Healthcare), with 1 M H_3_PO_4_ and 1 M NaOH as anode and cathode buffers, respectively. The pH gradient was measured directly on the gel using a contact electrode (Crison). β-glucosidase activity was detected after incubation of the gels with 2 mM *p*-methylumbelliferyl-β-d-glucopyranoside (Sigma-Aldrich), with fluorescence visualized under UV light by use of the Gel Doc XR + system (Bio-Rad).

The effect of pH on the BGLs was assessed using 100 mM Britton and Robinson buffer [44], which can be adjusted to a broad range of pH (2–10) Optimal pH of the pure BGLs was tested using *p*NPG as substrate in the same buffer, and to study their stability to pH, the samples were incubated from pH 2 to 10 for 72 h at 4 °C. The effect of temperature on enzymes activity was analyzed subjecting solutions of the pure enzymes in 100 mM sodium acetate buffer, pH 4 to temperatures between 30 and 80 °C for 10 min to determine their optimal temperature, and at 30 and 70 °C for 72 h to evaluate their thermostability.

Three dimensional models were generated by homology using the SWISS-MODEL server [45], based on sequence similarity. The QMEAN index is used to select the best models, which are ideal when the number is close to zero. The PyMol v0.99 [46] program was used to visualize and analyze the structures.

### Circular dichroism spectroscopy

Circular dichroism analysis of the native and recombinant BGL-2 forms were performed to compare their secondary structure. Measurements were carried out using a JASCO J-720 spectropolarimeter. Far-UV spectra (190–260 nm) were recorded in a 0.1 cm path length quartz cell at a protein concentration of 0.1 mg/mL in 10 mM phosphate buffer. The spectra from five scans were averaged and corrected for the baseline contribution of the buffer. The observed ellipticities were converted into mean residue ellipticities (*θ*) based on a mean molecular mass (per amino acid residue) of 110 Da.

### Protein quantification, enzyme assays and substrate specificity

In this study, all enzyme assays were performed in the presence of BSA (0.1%), a protein that does not affect the catalytic activity of the BGL but prevents missing activity when working at low enzyme concentrations [47].

Total protein was estimated by the bicinchoninic acid assay (BCA) method, using bovine serum albumin as standard. BGL activity was usually determined at 60 °C versus *p*-nitrophenyl-β-d-glucopyranoside (*p*NPG) in 50 mM acetate buffer pH 4, and 0.2% of BSA (standard assay). The reaction was stopped with 500 µL of sodium carbonate (2% w/v), and the release of *p*-nitrophenol (*p*NP) was measured in a spectrophotometer at 410 nm. One unit of BGL activity was defined as the amount of enzyme capable of hydrolyzing 1 μmol of *p*NPG to glucose and *p*NP per minute. The same conditions were used to measure hydrolysis of *o*-nitrophenyl-β-d-glucopyranoside (*o*NPG) and other nitrophenol derivatives.

BGLs were also incubated with different cellooligosaccharides. Its activity was determined against cellobiose, cellotriose, cellotetraose, cellopentaose, and cellohexaose, in 10 min-reactions carried out in sodium acetate 100 mM, pH 4, mixing at 1200 rpm. The released glucose was measured using the Glucose-TR (Spinreact) commercial kit, according to the manufacturer’s instructions.

The kinetic constants of the purified BGLs were determined by incubating the enzymes at their optimal pH and temperatures. The following substrates were analyzed over the range of concentrations stated in each particular case: *p*NPG (from 10 to 5 mM), *o*NPG (40–20 mM), cellobiose (80–40 mM), cellotriose (80–40 mM), cellotetraose (80–40 mM), cellopentaose (40–20 mM), and cellohexaose (20–10 mM). The values of *K*_m_ and *V*_max_ were determined from Lineweaver–Burk linear equation of the Michaelis–Menten model, using the program Sigmaplot.

The activity of BGL-2, BGL-2* and BGL-2T* was also determined against different polysaccharides, prepared in 50 mM sodium acetate buffer pH 4: 1.25% (w/v) Avicel (microcrystalline cellulose), 3% (w/v) Carboxymethyl cellulose (CMC), and 3% (w/v) beechwood xylan. The reaction mixture was incubated in a heating block at 1200 rpm for 10 min. After the incubation time, the reducing substances released was determined by the Somogyi–Nelson method [48], measuring A_540 nm_.

A control without enzyme was included in all assays, to substrate the absorbance due to reactants. Also, a negative control with inactivated (boiled 20 min) enzyme was included to discard unspecific interactions with substrates. Celluclast 1.5 L (Novozymes) was included as positive control in CMCase, Avicelase and xylanase assays.

### Cellulose binding assay

To evaluate the ability of native BGL-2 and its recombinant forms to bind microcrystalline cellulose and the participation of the CBD in this interaction, adsorption tests on Avicel were performed [49]. 20–30 ng of the purified enzymes were mixed with 500 µL of 1% Avicel (w/v) in 55 mM sodium acetate buffer pH 4. The reaction was carried out at 1200 rpm and 4 °C for 24 h. Aliquots were taken at different times (10 min, 1, 2, 3 and 24 h) after centrifuging the samples for 1 min at 14,000 rpm, before measuring residual BGL activity in the supernatants.

### Brewers spent grain saccharification

The efficiency of the different BGL-2 forms as β-glucosidase supplements for the saccharification of lignocellulosic residues was tested following the release of glucose from brewers spent grain. Mixtures of Celluclast 1.5 L and either purified BGL-2 enzymes or NS-50010, a commercial cocktail with high β-glucosidase activity, were used as catalysts. Brewers spent grain (100 mg) was treated with 2 mL of an enzymatic solution in 100 mM sodium acetate buffer, pH 4, containing a total of 2 BGL U/mL: 1 U/mL from Celluclast 1.5 L and 1 U/mL from the BGL sources compared (NS-50010 or purified BGL-2 enzymes). Reactions were performed in a heat block at 50 °C and 1200 rpm for 72 h. Free glucose was measured at different reaction times using the Glucose-TR commercial kit (Spinreact).

## Additional file

**Additional file 1: Table S1.** Comparison of the kinetic parameters reported or calculated for different fungal β-glucosidases using pNPG as model substrate. **Figure S1.** Sequence of *bgl2* from *T. amestolkiae*. **Figure S2.** MALDI-TOF mass spectra of BGL-2T* and BGL-2*. **Figure S3.** Protein sequence of BGL-2. **Figure S4.** Circular dichroism spectra (far UV spectrum) of purified BGL-2 isoforms. **Figure S5.** Cellulose-binding assays of BGL-2* against Xylan and Chitin, compared with Avicel.

## Abbreviations

BGL: β-1,4 glucosidase
MALDI-TOF: matrix-assisted laser desorption/ionization-time of flight
GH: glycosyl hydrolase
CMC: carboxymethyl cellulose
HPLC: high performance liquid chromatography
*p*NP: *p*-nitrophenol
CBD: cellulose binding domain
