# Supplementary material for: A novel, highly efficient β-glucosidase with a cellulose-binding domain: characterization and properties of native and recombinant proteins
Source: Biotechnol Biofuels. 2017 Nov 6;10:256. doi: 10.1186/s13068-017-0946-2 (PMC5674860; doi:10.1186/s13068-017-0946-2)
Supplement: Supplementary file 1 — Additional file 1: Table S1. Comparison of the kinetic parameters reported or calculated for different fungal β-glucosidases using pNPG as model substrate. Figure S1. Sequence of bgl2 from T. amestolkiae. Figure S2. MALDI-TOF mass spectra of BGL-2T* and BGL-2*. Figure S3. Protein sequence of BGL-2. Figure S4. Circular dichroism spectra (far UV spectrum) of purified BGL-2 isoforms. Figure S5. Cellulose-binding assays of BGL-2* against Xylan and Chitin, compared with Avicel. [file 13068_2017_946_MOESM1_ESM.docx]

Table S1. Comparison of the kinetic parameters reported or calculated for different fungal β-glucosidases using *p*NPG as model substrate.

| **Organism and reference** | **Enzyme** | ***K_m_*** | ***V_max_*** | ***k_cat_*** | ***k_cat_/K_m_*** |
| --- | --- | --- | --- | --- | --- |
| **Native BGLs** |  |  |  |  |  |
| *Trichoderma reesei* [49] | Cel3A | N | 41.0 |  |  |
|  | Cel3B | N | 36.0 |  |  |
|  | Cel1A | N | 2.9 |  |  |
| Metagenome from compost [50] | Td2f2 | 0.39 |  | 12.0 | 30.6 |
| *Thermoanaerobacterium thermosaccharolyticum [51]* |  | 0.63 | 64.0 | 55.4^a^ | 88.0^a^ |
| *Penicillium purpurogenum* [40] |  | 5.10 | 934.0 | 1395.1^a^ | 273.5^a^ |
| **BGLs expressed in *P. pastoris*** |  |  |  |  |  |
| *Thermoascus aurantiacus* [28] | BGLI | 0.22 | 71.7 |  |  |
| *Aspergillus fumigatus* [29] | rBgl3 | 1.76 | 131.4 | 284.8 | 161.7 |
| *Penicillium funiculosum* [32] | rBgl4 | 2.50 | 3332.0 | 7219.0^a^ | 2887.6^a^ |
| *Neurospora crassa* [33] | BGL2 | 0.21 | 143.2 | 181.2^a^ | 94.8^a^ |
| *Myceliophthora thermophila* [30] | MtBgl3a | 0.39 | 97.7 | 146.5^a^ | 375.7^a^ |
| *Saccharomycopsis fibuligera* and *T. reesei* [52] | SfBGL1/TrBGL1 | 0.20/0.21 |  | 7.2/10.6 | 34.8/50.2 |
| *Neosartorya fischeri* [31] | NfBGL1 | 0.51 | 2172.0 | 2853.0 | 5594.0 |
| **BGLs from this work** |  |  |  |  |  |
| ***T. amestolkiae*** | **BGL-2** | **0.41** | **299.7** | **485.8** | **1167.8** |
|  | **BGL-2*** | **0.19** | **261.1** | **444.1** | **2243.1** |
|  | **BGL-2T** | **0.34** | **649.1** | **874.0** | **2563.2** |

^a^Calculated from data provided in the original article. N means data is not given in original article.

Figure S1. Sequence of *bgl2* from *T. amestolkiae*. Introns are indicated in grey. The CBD coding region is represented after the third intron. Start and stop transcription codons are marked in bold. The predicted region for the signal peptide is underlined. The TAA stop codon present in third intron that prematurely ends transcription, producing BGL-2T*, is underlined and in bold grey.

Figure S2. MALDI-TOF mass spectra of BGL-2T* and BGL-2*.


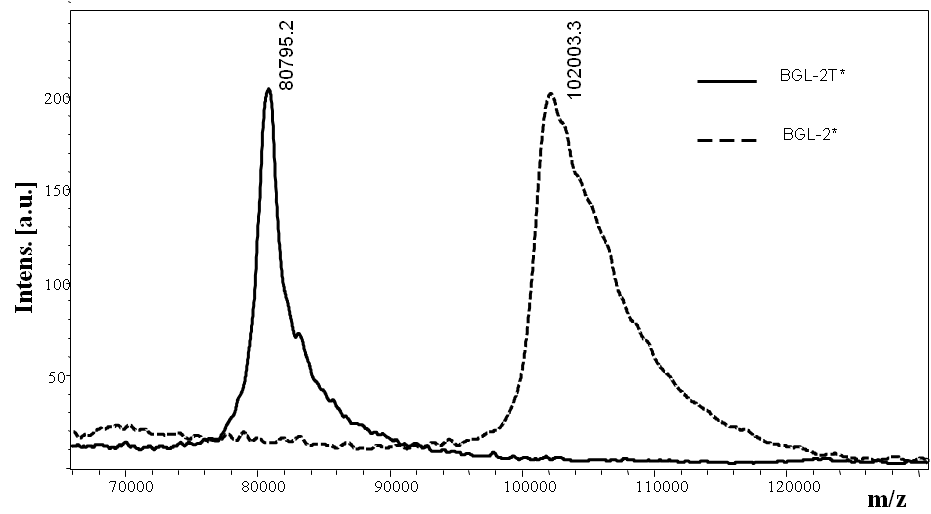


Figure S3. Protein sequence of BGL-2. The region underlined at the beginning corresponds to the predicted signal peptide, the catalytic residues are marked in black, the possible glycosylation sites are shown in grey. The missing residues in BGL-2T* are indicated in italics. CBD of the complete form is highlighted inside a rectangle.

Figure S4. Circular dichroism spectra (far UV spectrum) of purified BGL-2 isoforms.

Figure S5. Cellulose-binding assays of BGL-2* against Xylan and Chitin, compared with avicel.
